# Appendicitis as an Early Manifestation of Subsequent Malignancy: An Asian Population Study

**DOI:** 10.1371/journal.pone.0122725

**Published:** 2015-04-27

**Authors:** Shih-Chi Wu, William Tzu-Liang Chen, Chih-Hsin Muo, Fung-Chang Sung

**Affiliations:** 1 Trauma and Emergency Center, China Medical University Hospital, Taichung 404, Taiwan; 2 College of Medicine, China Medical University, Taichung 404, Taiwan; 3 Division of Colorectal Surgery, Department of Surgery, China Medical University Hospital, Taichung 404, Taiwan; 4 Management Office for Health Data, China Medical University and Hospital, Taichung 404, Taiwan; 5 Institute of Clinical Medical Science, China Medical University College of Medicine, Taichung 404, Taiwan; Institute of Psychiatry, UNITED KINGDOM

## Abstract

**Background & Aims:**

Cancer risk after appendectomy in patients with appendicitis remains unclear. This study examined the role of appendicitis as an early manifestation harbingering the distant malignancy.

**Methods:**

From the insurance claims data of Taiwan, we identified a cohort of 130,374 patients newly received appendectomy from 2000–2009, without cancer diagnosis. A comparison cohort of 260,746 persons without appendectomy and cancer was selected from the same database, frequency matched by age, sex, comorbidity and index year. We monitored subsequent cancers with a12-month follow-up.

**Results:**

Over all, 1406 and 616 cancer cases were identified in the appendectomy cohort and comparisons, respectively, with all cancers incidence rate 4.64-fold higher in the appendectomy cohort (9.06 vs. 1.96 per 1000 person-months). Digestive and female genital organs harbored 80.9% of cancer cases in the appendectomy cohort. The Cox model measured site-specific hazard ratio (HR) was the highest for female genital cancers (23.3), followed by cancers of colorectum (14.7), small intestine (10.1), pancreas (7.40), lymphoma (5.89) and urinary system (4.50), all significant at 0.001 level. The HR of all cancers decreased from 13.7 within 3 months after appendectomy to 1.37 in 7–12 months after the surgery. In general, relative to the comparison cohort, younger appendectomy patients tended to have a higher HR than older patients.

**Conclusions:**

The high incident cancers identified soon after appendectomy suggest the acute appendicitis is the early sign of distant metastatic malignancy. The risk of colorectal cancer, female genital cancer and haemopoietic malignancy deserve attention.

## Introduction

Appendectomy is the most common cause of emergent abdominal surgery for appendicitis with a lifetime risk of near 7.0% [1]. The etiology of appendicitis has been attributed to the luminal obstruction of the appendix and the inflammation of the appendix^1^. Earlier case-control studies found that patients received appendectomy are at increased risks of cancers, such as breast and uterine cancers [2–5]. These studies were limited to small samples for assessment. A Swedish cohort study shows that children with appendectomy are at an excess risk of stomach cancer and non-Hodgkin’s lymphoma [6]. A Danish study followed 82,000 patients who had received appendectomy because of appendicitis failed to achieve similar results. Relative to the Danish Hospital Discharge Register, these appendectomized patients have a standardized incidence ratio of 1.05 (95% confidence interval 0.99–1.11) for the total number of malignancy [7].

Evidences on appendectomy in the etiology of subsequent cancer risk remains unclear [6–11]. Case studies have associated acute appendicitis with prostate cancer as the primary symptom [8], recurrent gastric adenocarcinoma [9], and the first manifestation of colon cancer [10] and leukemia [11]. These studies suggest that the cancers may metastasize to appendix resulting inflammation. It is necessary to conduct epidemiologic studies using large samples to determine whether appendicitis is associated with the risk of cancer. Claims data of Taiwan National Health Insurance (NHI) based on a large population size provides an opportunity to evaluate this relationship.

We were interested in investigating the presentation of various types of cancer for patients who had received appendectomy, hypothesizing appendicitis as an early sign associated with malignancy development. Appendectomy is the mainstay of treatment for patients suffering from acute appendicitis. We therefore established a cohort of patients who had received surgery because of appendicitis and a comparison cohort without the history of appendicitis to compare the risk of malignancy by site. We also estimated the site-specific malignancy hazards between men and women and among age groups, focusing on the malignancy trend during a 12-month follow-up period after appendectomy. In this study, appendicitis and appendectomy are interchangeable words.

## Methods and Materials

### Data source

The NHI of Taiwan is a single-payer insurance program, covering over 99.5% of population and contracting 92% of hospitals in Taiwan as of 2010. This retrospective follow-up study used inpatient claims data and the registries of catastrophic illness patients, in the period of 1996–2010, obtained from the National Health Research Institutes, which has been responsible for managing the claims data with the authorization of the Bureau of National Health Insurance. All cancer diagnoses were verified histologically and/or at least twice typical radiological image examinations by ultrasound, contrast-enhanced dynamic computed tomography or magnetic resonance imaging. Patient records registered in data files were linked using encrypted identification numbers of insured people to fulfill the Personal Information Protection Act. We conducted this study with the approval of the Ethics Review Committee at Chinese Medical University and Hospital.

Although written informed consent was not provided by the participants for the use of their clinical records in this study, the patient records/information was anonymized and de-identified prior to the analysis.

### Study subjects

**Fig 1:**
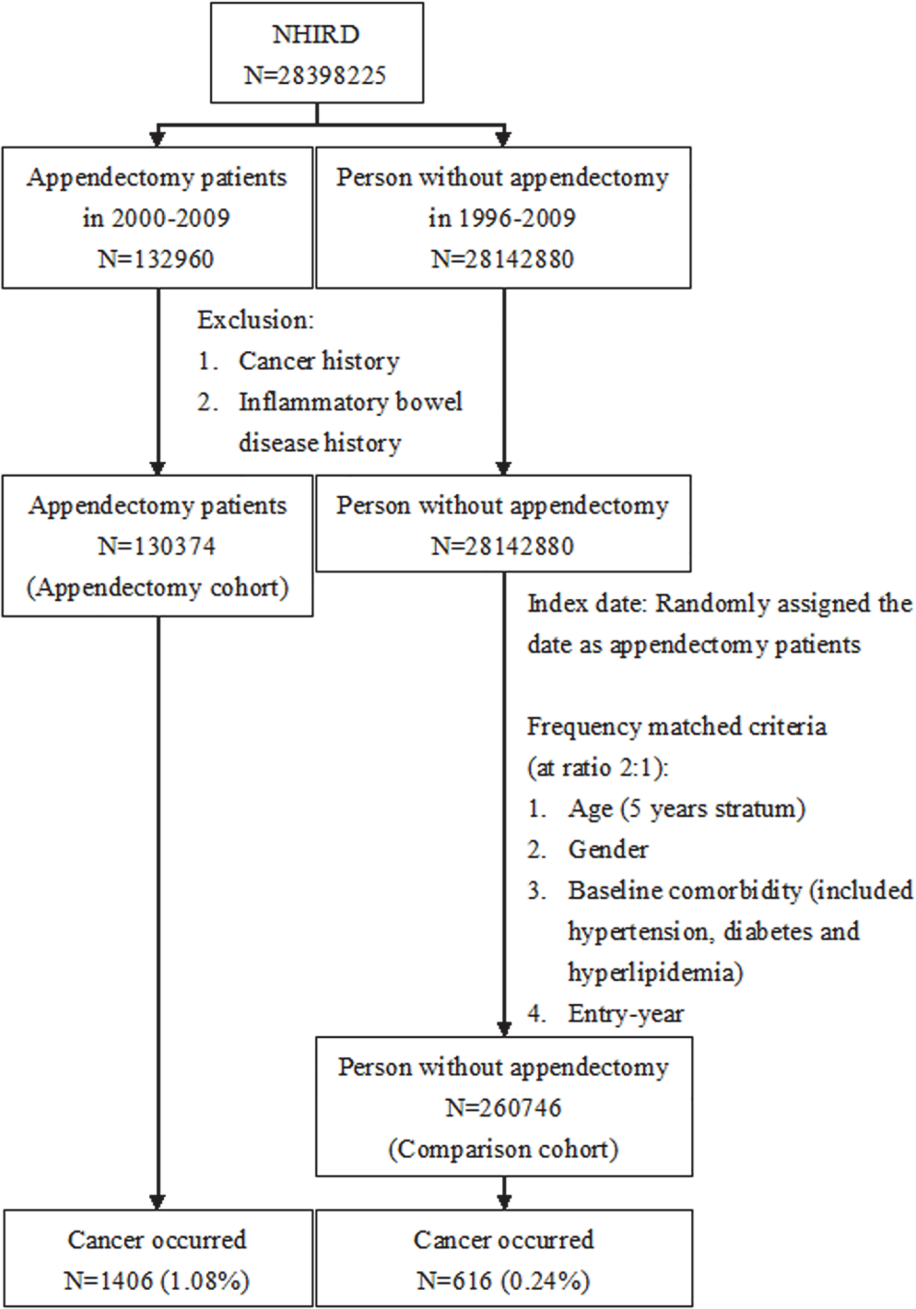
Flow chart for study subjects.

For determining the appendectomy cohort, we identified 132960 patients with appendicitis and newly received appendectomy [The International Classification of Diseases, 9th Revision, Clinical Modification (ICD-9-CM) Operation code: 47.0, 47.01and 47.09] in 2000–2009 from inpatient claims. Patients with the history of malignancy (ICD-9-CM code: 140–208) and/or inflammatory bowel disease (ICD-9-CM 555.00–555.02, 555.9 and 556) at least 4 years before appendectomy, and missing information of gender or age, were excluded. We also excluded appendectomy patients who had a history of malignant metastasis (ICD-9-CM code: 196–199) or appendix malignancy (ICD-9-CM code: 153.5) before the date of appendectomy. The remaining 130374 patients were included in the appendectomy cohort and the date for appendectomy was defined as entry date (Fig 1).

For each appendectomy case selected, two comparison subjects without the history of cancer and inflammatory bowel disease, in the past 4 years at least, were randomly selected and assigned the entry date as appendectomy patients. They were frequency matched by age at entry date (5 years stratum), gender, entry-year and baseline comorbidities. Baseline comorbidities including hypertension (ICD-9-CM code: 401–405), diabetes (ICD-9-CM code: 250) and hyperlipidemia (ICd-9-CM: 272) were identified from inpatient claims and defined since 1996 to entry date. Comparison subjects who had developed malignant metastasis or appendix malignant (ICD-9-CM code: 153.5) within one year after being selected in the cohort were also excluded.

### Statistical analysis

Data analysis first illustrated the distributions of demographic status and comorbidities and compared between the two cohorts. This study attempted to identify the short term cancer risk for the appendectomy cohort. Study subjects were followed from the entry dates to the date with first cancer diagnosed, censored because of loss to follow-up or withdraw from the insurance within 12 months. The person-months follow-up time was thus counted for each subject with a maximum of 12-month follow-up period. The cancer type was identified based on the first cancer diagnosis. The incidence of malignancy (per 1000 person-months) was calculated by the cancer site between two cohorts. The Cox proportional hazards regression analysis was used to calculated hazard ratio (HR) and 99.9% confidence intervals (CIs) of malignancy for the appendectomy cohort compared with the comparison cohort, based on the Bonferroni adjustment for multiple comparisons at the 2-tailed test. The data analysis also assessed age- and gender-specific risks. This study violated the assumption of Cox proportional hazard regression according to testing the association between Schoenfeld residuals for appendectomy and follow-up time (p<0.0001). We, therefore, estimated the HRs of all cancers and by the site in the follow-up for periods of ≤ 3 months, 4–6 months and 7–12 months to illustrate the trend of cancer development. All statistical analyses were performed using SAS version 9.3 (SAS Institute Inc.).

## Results

The study population consisted of 130374 appendectomy patients and 260746 comparison subjects in this study. The descriptive information in Table 1 shows both cohorts had similar distributions of sex, age and comorbidities, prone with men (53.2%) and subjects < 40 years-old (68.3%) with a mean age of 33.3 years (standard deviation = 18.1). The prevalence rates of comorbidities were low for hypertension (5.05%), diabetes (2.96%) and hyperlipidemia (1.29%) in the study populations.

**Table 1:** Distribution in demographics and comorbidities compared between two cohorts.

		Appendectomy		Comparison		
		N = 130374		N = 260746		
Variable		n	%	n	%	p-value
Sex	Men	69358	53.2	138714	53.2	0.96
	Women	61016	46.8	122032	46.8	
Age, year	< 20	34073	26.1	68144	26.1	0.95
	20–39	54999	42.2	109998	42.2	
	40–59	28470	21.8	56940	21.8	
	60–79	11354	8.71	22708	8.71	
	≥ 80	1478	1.13	2956	1.13	
	Mean (SD)	33.3	(18.1)	33.3	(18.1)	0.88
Baseline comorbidity	Hypertension	6581	5.05	13160	5.05	0.96
	Hyperlipidemia	1682	1.29	3362	1.29	0.90
	Diabetes	3861	2.96	7720	2.96	0.95

SD, standard deviation.

**Fig 2:**
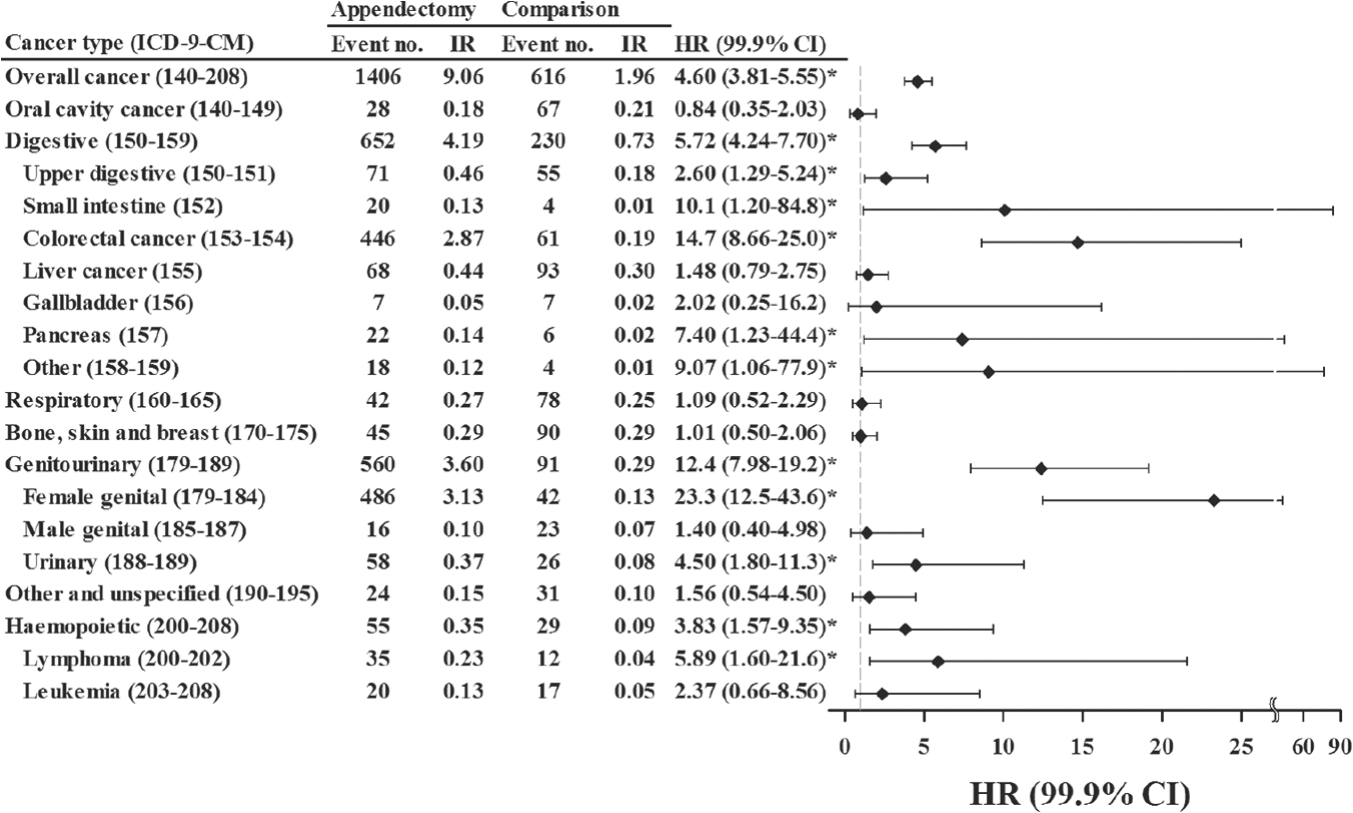
Incidence of cancer in study cohorts and Cox methods measured hazard ratios and 99.9% confidence interval of cancer associated with appendectomy in by cancer site.

Fig 2 shows a total of 1406 and 616 cancer events developed in the appendectomy cohort and the comparison cohort, respectively, during the follow-up period. The incidence of all cancers was 4.60-fold higher in the appendectomy cohort than in the comparison cohort (9.06 vs. 1.96 per 1000 person-months) with a HR of 4.60 (99.9% CI = 3.81–5.55) for patients with appendectomy. Most of organ-specific malignant incidences in appendectomy patients were identified for digestive organs (n = 652, or 46.3%) and followed by genitourinary organs (n = 560, or 39.8%) and significant for haemopoietic system (n = 55, or 3.9%). The genitourinary cancers were mainly female genital cancer (HR = 23.3, 99.9% CI = 12.5–43.6). Among the digestive organs, the highest hazard was identified for colorectal cancer (HR = 14.7, 99.9% CI = 8.66–25.0), followed by small intestine cancer (HR = 10.1, 99.9% CI = 1.20–84.8) and other sites (HR = 9.07, 99.9% CI = 1.06–77.9). Patients received appendectomy also had increased incidence of lymphoma with a HR of 5.89 (99.9% CI = 1.60–21.6).

Table 2 shows the gender-specific HR of cancer for the appendectomy cohort compared to the comparison cohort by cancer site. The HR of all cancers was greater for women than for men. The site-specific data also show that women also had higher HR of cancers than men for genitourinary cancer and haemopoietic cancer, while men were at higher hazard for colorectal cancer. Table 3 shows the age-specific HRs of cancers for the appendectomy patients relative to comparisons by site. The HR decreased by age for cancers of all sites and of most sites. However, women with appendectomy 45–64 of ages had a higher HR of genitourinary cancer.

**Table 2:** Cox method measured hazard ratio and 99.9% confidence interval of cancer associated with appendectomy by cancer site and gender.

	Women	Men
Cancer type (ICD-9-CM)	HR (99.9% CI)	HR (99.9% CI)
Overall cancer (140–208)	5.91 (4.53–7.70)*	3.46 (2.64–4.53)*
Oral cavity cancer (140–149)	0.41 (0.02–8.24)	0.92 (0.37–2.31)
Digestive (150–159)	6.13 (3.73–10.1)*	5.49 (3.78–7.97)*
Upper digestive (150–151)	3.84 (0.84–17.5)	2.33 (1.06–5.14)*
Small intestine (152)	4.54 (0.44–47.1)	—
Colorectal cancer (153–154)	12.3 (5.57–27.2)*	16.8 (8.19–34.3)*
Liver cancer (155)	1.60 (0.54–4.75)	1.42 (0.67–3.02)
Gallbladder (156)	1.52 (0.08–29.6)	2.69 (0.14–52.5)
Pancreas (157)	7.40 (0.59–93.3)	7.39 (0.59–93.1)
Other (158–159)	6.72 (0.52–87.1)	16.1 (0.26–998)
Respiratory (160–165)	1.15 (0.35–3.73)	1.05 (0.40–2.75)
Bone, skin and breast (170–175)	0.91 (0.42–1.97)	2.02 (0.29–14.1)
Genitourinary (179–189)	18.4 (10.6–32.0)*	3.19 (1.40–7.31)*
Female genital (179–184)	23.3 (12.5–43.6)*	—
Male genital (185–187)	—	1.40 (0.40–4.98)
Urinary (188–189)	2.64 (0.63–11.1)	6.36 (1.84–21.9)*
Other and unspecified (190–195)	1.42 (0.37–5.50)	1.83 (0.34–10.0)
Haemopoietic (200–208)	4.85 (1.12–21.0)*	3.29 (1.06–10.2)*
Lymphoma (200–202)	4.71 (0.71–31.5)	7.06 (1.17–42.7)*
Leukemia (203–208)	5.05 (0.51–50.5)	0.30–7.96)

* p < 0.0001

**Table 3:** Cox method measured hazard ratio and 99.9% confidence interval of cancer associated with appendectomy by cancer site and age.

	< 45 years	45–64 years	≥ 65 years
Cancer type (ICD-9-CM)	HR (99.9% CI)	HR (99.9% CI)	HR (99.9% CI)
Overall cancer (140–208)	5.45 (3.69–8.08)*	4.92 (3.64–6.65)*	3.96 (2.92–5.38)*
Oral cavity cancer (140–149)	0.69 (0.17–2.89)	0.89 (0.24–3.22)	1.30 (0.14–12.0)
Digestive (150–159)	7.73 (3.34–17.9)*	5.53 (3.46–8.84)*	5.54 (3.58–8.57)*
Upper digestive (150–151)	4.59 (0.79–26.8)	2.27 (0.79–6.49)	2.46 (0.80–7.58)
Small intestine (152)	—	—	4.14 (0.38–44.9)
Colorectal cancer (153–154)	19.5 (4.16–91.2)*	19.3 (7.38–50.7)*	11.8 (5.83–23.8)*
Liver cancer (155)	2.68 (0.48–14.9)	1.36 (0.53–3.51)	1.40 (0.54–3.61)
Gallbladder (156)	2.01 (0.04–98.4)	1.53 (0.08–29.9)	4.16 (0.04–488)
Pancreas (157)	4.02 (0.03–472)	12.2 (0.18–818)	7.27 (0.80–66.0)
Other (158–159)	4.02 (0.03–471)	5.40 (0.39–75.2)	—
Respiratory (160–165)	2.01 (0.08–48.2)	0.90 (0.22–3.67)	1.17 (0.47–2.93)
Bone, skin and breast (170–175)	0.75 (0.20–2.81)	1.07 (0.40–2.84)	1.57 (0.28–8.73)
Genitourinary (179–189)	17.9 (7.45–42.8)*	22.5 (9.47–53.5)*	5.24 (2.68–10.2)*
Female genital (179–184)	20.1 (7.87–51.2)*	27.9 (10.2–76.4)*	22.2 (4.78–103)*
Male genital (185–187)	2.01 (0.01–492)	4.05 (0.04–475)	1.30 (0.33–5.15)
Urinary (188–189)	5.02 (0.19–130)	9.35 (1.37–63.8)*	3.28 (1.05–10.3)*
Other and unspecified (190–195)	1.88 (0.44–7.96)	1.53 (0.19–12.5)	1.05 (0.10–11.4)
Haemopoietic (200–208)	8.03 (1.55–41.6)*	2.82 (0.57–14.3)	2.26 (0.48–10.7)
Lymphoma (200–202)	17.1 (0.93–313)	3.74 (0.52–26.9)	3.65 (0.32–41.8)
Leukemia (203–208)	4.42 (0.54–36.0)	1.53 (0.08–29.9)	1.57 (0.19–12.8)

* p < 0.0001

The increased HR of developing cancer appeared soon after the surgery of appendectomy. Table 4 shows the HR of malignant development in the appendectomy cohort relative to the comparison cohort was the highest in the first 3 months post the surgery, and declining by the follow-up months. The HR of colorectal cancer declined from 45.9 (95% CI = 17.0–124) in the earlier 3 months post the surgery to 2.69 (95% CI = 1.10–6.60)7–12 months after the surgery. The corresponding HRs of female genital cancer in these 2 follow-up periods were 91.5 (95% CI = 28.4–317) and 1.46 (95% CI = 0.44–4.84), respectively.

**Table 4:** Cox method measured hazard ratios and 99.9% confidence interval of cancer associated with appendectomy by cancer site and follow-up months.

	≤3 months	4–6 months	7–12 months
Cancer type (ICD-9-CM)	HR (99.9% CI)	HR (99.9% CI)	HR (99.9% CI)
Overall cancer (140–208)	13.7 (9.84–19.1)*	1.56 (0.97–2.50)	1.37 (0.96–1.95)
Oral cavity cancer (140–149)	0.71 (0.13–3.90)	0.40 (0.04–4.74)	1.14 (0.36–3.58)
Digestive (150–159)	15.0 (9.06–24.9)*	2.51 (1.20–5.25)*	1.61 (0.93–2.79)
Upper digestive (150–151)	5.52 (1.94–15.7)*	1.21 (0.13–9.84)	1.05 (0.29–3.77)
Small intestine (152)	34.2 (0.63–1875)	—	3.03 (0.09–1.6)
Colorectal cancer (153–154)	45.9 (17.0–124)*	6.24 (1.71–22.7)*	2.69 (1.10–6.60)*
Liver cancer (155)	2.83 (1.02–7.83)*	1.29 (0.34–4.87)	0.84 (0.29–2.41)
Gallbladder (156)	4.04 (0.03–474)	0.51 (0.01–39.2)	4.04 (0.14–117)
Pancreas (157)	28.2 (0.50–1584)	3.03 (0.09–106)	3.37 (0.20–57.7)
Other	7.55 (0.85–67.4)	—	—
Respiratory (160–165)	2.02 (0.49–8.35)	0.70 (0.14–3.47)	0.96 (0.33–2.84)
Bone, skin and breast (170–175)	1.10 (0.27–4.45)	1.05 (0.29–3.78)	0.94 (0.32–2.76)
Genitourinary (179–189)	53.2 (21.5–132)*	1.93 (0.57–6.50)	1.47 (0.63–3.40)
Female genital (179–184)	91.5 (28.4–317)*	3.76 (0.61–23.3)	1.46 (0.44–4.84)
Male genital (185–187)	4.71 (0.32–69.0)	0.34 (0.01–22.5)	1.16 (0.21–6.48)
Urinary (188–189)	13.8 (2.52–75.4)*	1.52 (0.19–12.4)	1.85 (0.37–9.40)
Other and unspecified (190–195)	2.02 (0.35–11.5)	1.01 (0.15–7.07)	1.80 (0.27–11.9)
Haemopoietic (200–208)	11.7 (2.11–65.6)*	1.80 (0.27–11.9)	1.73 (0.38–8.00)
Lymphoma (200–202)	14.1 (1.28–156)*	1.69 (0.16–17.8)	6.06 (0.45–81.1)
Leukemia (203–208)	9.39 (0.79–112)	2.02 (0.08–48.5)	0.55 (0.04–6.95)

* p < 0.0001

## Discussion

Limited studies based on small samples have reported that patients with appendicitis receiving emergency surgery may have an elevated risk of cancers [2–5, 8–11]. The large Swedish study followed up an appendectomy cohort of children for a mean length of 11.2 years and found excess stomach cancer [6]. The Demark large cohort study of all ages failed to find this relationship [7].

In the present study, we found 1.08% of patients with appendicitis developed malignancy within 12 months after having appendectomy. The hazard was particularly high in the first 3 months after the surgery, indicating the development of cancers is a rapid process. The most eminent sites of malignancy were female genital organ (ovary and uterine), colorectum and small intestine and hematopoietic system (lymphoma and leukemia). It takes a long latent period for these tumors to develop. These cancers appearing in a short period is a subject of speculation as previously existing but unveiled by the presentation of appendicitis; while the patients of appendicitis with known coexisted metastatic malignancy has been carefully excluded in the process of establishing the study cohorts. The short-term relationship between appendectomy for acute appendicitis and cancer incidence was not specific observed in both Demark and Swedish studies. On the other hand, we measured only the short-term outcome with strong relationship for a few cancer sites. Therefore, appendicitis is possible an early manifestation of undetected preexisting cancers.

Based on the substantial association with lymphatic tissue, recent studies have proposed that the human vermiform appendix carries out immune functions, instead of is an evolutional vestige structure of functionless [12,13]. Inflammation of the appendix is common because of the obstruction of the appendiceal lumen by fecalith, parasites, lymphoid hyperplasia, or sometimes by neoplasms [10]. The infiltration inflammatory change of the lymphoid tissue in the appendix can also lead to luminal obstruction and acute superimposed infection [14].

There are no clear empirical evidences to explain the relationship appears as a harbinger phenomenon in our study. However, the appendix shares the same lymphatic system named gut associated lymphatic tissue (GALT) of the intestine [13]. As of colorectal cancers, it would be rational to hypothesize that the tumor cells may metastasize to appendix via the early lymphatic drainage system, resulting the inflammation of the appendix. In addition, the colorectal cancer can be spread by hematogenous and lymphatic route, as well as adjacent or transperitoneal dissemination. Common metastatic sites have been reported for the regional lymph nodes, liver, lungs, and peritoneum [15]. The tumor invasion metastasis may cascade and arrest at a distal organ site after survival in the circulation [16]. The appendiceal vessel is at the terminal location under the confluence of small bowel and large intestine. Tumor cells may intravasate into blood vessel to be arrested in the conjunction, resulting early inflammation and presenting with clinically appendicitis in the early stage of tumor metastasis, which might be undetected at pathologic examination.

The architecture of appendix has been considered as a “safe house” with the function of providing support for bacterial growth [17, 18], Removal of the appendix (often in the situation of appendicitis) may result in imbalanced colon microbiota. Among these bacterial species, *Fusobacterium* species are responsible for the majority of cases of acute appendicitis [19,20], and may link appendicitis to subsequent inflammatory bowel diseases and colorectal cancer [21–24]. Patients receiving appendectomy because of appendicitis may be at a risk for subsequent colorectal cancer. However, further clinical or basic studies are needed to evaluate whether appendicitis/appendectomy is a risk factor, a symptom, or a comorbidity of cancers.

The incidences of gall bladder cancer and pancreatic cancer after the surgery of appendicitis were also elevated for 2.02-fold and 7.40-fold, respectively, in the present study (Fig 2), even in aged patients although not significant (Table 3). In general, the age-standardized incidences of gallbladder and pancreatic cancer are 2.53 and 5.62 per 100,000 person, respectively, in Taiwan [25]. The incidence of gall bladder cancer is relatively lower and is around 1.2 cases per 100,000 population per year in United States [26], whereas pancreatic cancer is reported the fourth most common cause of cancer mortality in United States, with lower 5-year survival rates [27]. Nonetheless, there was no report investigated the relationship between appendicitis and gall bladder and pancreas malignancies. It is of interest that whether the tumor cells may also intravasate into blood vessel to be arrested in the conjunction of appendix [16]; or aged appendicitis may simply represented an outcome of synchronized declination of the digestive system, including hepatobiliary system and intestine, This hypothesis need further studies.

In the present study, the cancer risk was much greater for female genital organs (ovary and uterus) and urinary organ (bladder) in 12 months after the surgery of appendicitis. The appendix and bladder and female genital organs are generally located in the intra-pelvic cavity. It would be rational to assume that the adjacent or transperitoneal dissemination of metastasis through local invasion results appendicitis. It is likely to occur in stage II-III of tumor metastasis [16].

It is uncommon to note the leukemic and lymphomatous infiltration of the appendix associated with haemopoietic malignancy. The first description can be traced to 1967 by Rappaport [28]. Few case studies have reported later considering acute appendicitis as the early presentation of leukemia and lymphoma [29]. Other case reports also considered the nonsuppurative appendicitis as the initial manifestation of acute myelogenous leukemia [30]. A recent study linked acute appendicitis to acute myeloid leukemia [31]. Our study showed a strong relationship between appendicitis and haemopoietic malignancy, with an HR of 11.7 for the cancer to be diagnosed within 3 months after the surgery. Karachiwala et al. recently reported a young men with acute promyelocytic leukemia developing acute appendicitis on the 11th day of chemotherapy for the cancer [32]. The leukemic infiltration could be accountable for the development of acute appendicitis in the present study as well.

### Limitation of the study

This study has the advantage of using a large population data with reliable diagnosis, allowing a longitudinal observation with a low loss to follow-up rate. Certain limitations do exist. First, life style information regarding drinking, smoking, diet, and genetic factors, etc. are not all available for the cancer risk adjustment. The prevalence of smoking is much lower in women than in men (4.3 vs. 46.8%) in Taiwan [33]. However, in the present study, the HR of all cancers post the surgery was even greater for women than men, and the HRs of respiratory cancer were low for both women and men. Thus, the life style factors are less likely to affect the estimated results. Second, despite of the meticulous study design, bias resulting from retrospective nature may have occurred due to unmeasured comorbidities. Third, all data used are anonymous. Therefore, relevant clinical variables, such as pathology findings, imaging results, and serum laboratory data are unavailable in our study. However, the data regarding appendectomy, and cancer diagnoses were highly reliable.

## Conclusion

Our findings reveal a strong risk of subsequent cancer for patients having an appendectomy. Appendicitis could be an early manifestation or harbinger of subsequent malignancy risk. Routine follow up programs for appendectomy patients should be considered to screen for specific malignancy. This is especially important for colorectal cancer in both men and women and for genital cancer and haemopoietic malignancy in women. Earlier detection and early treatment of subsequent cancer may benefit these patients.
